# Fibroblast Growth Factor 9 as a Potential Biomarker for Schizophrenia

**DOI:** 10.3389/fpsyt.2022.788677

**Published:** 2022-04-25

**Authors:** Xiao-Ling Li, Yun Yu, Yang Hu, Huan-Tong Wu, Xue-Song Li, Guang-Yang Chen, Yong Cheng

**Affiliations:** ^1^The Third People’s Hospital of Foshan, Foshan, China; ^2^Center on Translational Neuroscience, College of Life and Environmental Sciences, Minzu University of China, Beijing, China

**Keywords:** fibroblast growth factor 9, schizophrenia, antipsychotics, biomarker, first-episode, drug-free

## Abstract

Preclinical and clinical studies have suggested that fibroblast growth factor (FGF) system contributed to the onset and development of schizophrenia (SCZ). However, there was no strong clinical evidence to link an individual FGF with SCZ. In this study, we aim to measure blood FGF9 levels in the patients with SCZ with and/or without medication, and test whether FGF9 has a potential to be a biomarker for SCZ. We recruited 130 patients with SCZ and 111 healthy individuals, and the ELISA and qRT-PCR assays were used to measure serum FGF9 levels in the participants. ELISA assay demonstrated that serum FGF9 protein levels were dramatically reduced in first-episode, drug-free patients, but not in chronically medicated patients when compared to healthy control subjects. Further analysis showed that treatment of the first-episode, drug-free SCZ patients with antipsychotics for 8 weeks significantly increased the serum FGF9 levels. In addition, we found that blood *FGF9* mRNA levels were significantly lower in first-onset SCZ patients than controls. Under the receiver operating characteristic curve, the optimal cutoff values for FGF9 protein level as an indicator for diagnosis of drug-free SCZ patients was projected to be 166.4 pg/ml, which yielded a sensitivity of 0.955 and specificity of 0.86, and the area under the curve was 0.973 (95% CI, 0.954-0.993). Furthermore, FGF9 had good performance to discriminate between drug-free SCZ patients and chronically medicated patients, the optimal cutoff value for FGF9 concentration was projected to be 165.035 pg/ml with a sensitivity of 0.86 and specificity of 0.919, and the AUC was 0.968 (95% CI, 0.944, 0.991). Taken together, our results for the first time demonstrated the dysregulation of FGF9 in SCZ, and FGF9 has the potential to be served as a biomarker for SCZ.

## Introduction

Schizophrenia (SCZ) is a chronic, serious psychosis with an incidence of approximately 1% (1). The clinical manifestations are mainly emotional reactions characterized by paranoia, hallucinations and inappropriate social misconduct (2). The mechanism underlying the devastating disease is very complex and still poorly understood, although it is considered that the occurrence of the disease has a very strong relationship with genetic and the environment background (3). More recently, there is increased awareness of the critical role of epigenetics in the onset and/or development of SCZ (4, 5). The typical treatments for SCZ are antipsychotics which target the neurotransmitter system in the central nervous system (6). However, long-term use of these drugs had side effects, and failed to improve the negative symptom and cognitive impairment of the patients (7, 8). In addition, it is often difficult to distinguish SCZ with other neuropsychiatric diseases due to the lack of the specific biomarkers. Therefore, there is an urgent need to better understand the etiology of SCZ and subsequently develop biomarkers for the diagnosis and prognosis of the disease.

The neurotrophic factor hypothesis of schizophrenia has generated great interest over the last decade. It postulated that the disturbances of developing processes involving neurotrophic factors result in the changes in the brain of patients with SCZ (9). This hypothesis was mainly supported by the large number of preclinical and clinical studies suggesting the involvement of brain-derived neurotrophic factor and nerve growth factor in the pathogenesis of SCZ (10–13). More recent studies suggested that fibroblast growth factor system is involved in the pathogenesis of SCZ. FGF2 is the most studied FGFs in the literature, which has been demonstrated to play crucial roles in neurodevelopment and maintenance of nervous system in adult (14, 15). The clinical data from Hashimoto et al. showed that blood FGF2 levels were significantly increased in the medicated patients with SCZ when compared with healthy controls, but they did not show a statistically significant difference between non-medicated patients and controls for FGF2 levels (16). In contrast, results from Li et al. indicated non-medicated SCZ patients had higher serum FGF2 levels than healthy individuals (17). In addition to FGF2, FGF9 has been proposed as a novel modulator for mood disorder, as both preclinical and clinical data indicated a role of FGF9 in depression (18, 19). Moreover, FGF9 has been suggested to be involved in the development of SCZ, this is supported by the impaired social discrimination and heightened acoustic startle reactivity in FGF9 mutant mice (20). However, there is no clinical data to support a role of FGF9 in SCZ.

In this study, we recruited 130 SCZ patients and 111 healthy control (HC) subjects to determine whether FGF9 levels were dysregulated in the peripheral blood of SCZ patients. Our results indicated that the levels of FGF9 in peripheral blood of SCZ patients were significantly reduced, and treatments with the antipsychotics restored the FGF9 levels in the patients. Our analyses also suggested that serum FGF9 has the potential to be served as a biomarker to inform the diagnosis and/or treatment response for SCZ.

## Materials and Methods

### Subjects

We recruited 130 SCZ patients from The Third People’s Hospital of Foshan. SCZ Patients diagnosed by experienced psychiatrists according to Structured Clinical Interview for DSM-IV (SCID) and International Classification of Diseases 10 (ICD-10) were included, including 57 patients who were first-episode, drug-free SCZ patients, and 73 patients with chronic medication. It should be noted that 19 patients from the group of drug-free SCZ patients had two evaluations: baseline and after 8-week antipsychotics treatments. Positive and negative syndrome scale (PANSS) was used to assess the patients’ psychiatric symptoms. All patients with intellectual disability, severe physical illnesses, substance abuse, and serious infectious diseases were excluded. At the same time, 111 healthy volunteers without mental and physical illnesses were recruited as controls.

All subjects included in this study have signed an informed consent form (or by family members for some patients). The research plan was reviewed and approved by the Ethics Committee of the Third People’s Hospital of Foshan, Guangdong, China. And the experiments were conducted in accordance with the Helsinki Declaration.

### Enzyme-Linked Immunosorbent Assay

In total, 10 ml of peripheral blood from the patients or healthy individuals were collected in the morning after overnight fasting. The blood samples were allowed to clot at room temperature for 1 h, and then we obtained the serum by centrifugation at 3000 × *g* for 10 min. The serum was then stored in a −80 refrigerator for subsequent experiments. We used the enzyme-linked immunosorbent assay (ELISA) kit to measure FGF9 levels in serum. The specific protocol was carried out according to the instructions of the kit (Cloud-Clone, Wuhan, China; Catalog No: SEA036Hu). The sensitivity of the human serum FGF9 kit was 6.6 pg/ml, and the concentration of the protein was expressed as pg/ml serum.

### Measurement of Blood Fibroblast Growth Factor 9 Gene Expression

Blood *FGF9* mRNA levels of 48 first-onset SCZ patients and 48 HC subjects were measured by quantitative real time PCR (qRT-PCR). RNA extraction and reverse transcription were performed according to the manufacturer’s instruction (Beijing Zhuangmeng International Biological Gene Technology Co., Ltd, Beijing, China). qRT-PCR was performed as previously described (21), and the cycling conditions were: preincubation at 95°C for 600 s, followed by 45 cycles of synthesis at 95°C for 15 s, 59°C for 20 s and 72°C for 40 s. The primer sequences for FGF9 forward: 5′-ATGGCTCCCTTAGGTGAAGTT-3,′ reverse: 5′-CC CAGGTGGTCACTTAACAAAAC-3′; for GAPDH forward: 5′-CTGGGCTACACTGAGCACC-3,′ reverse: 5′-AAGTGGTCG TTGAGGGCAATG-3.′

### Statistical Analysis

All data were presented in the form of mean ± standard deviation (SD). We used GraphPad Prism 8.0 to calculate and graph the statistics for FGF9 expression analysis. The Kolmogorov-Smirnov test (normality test) was applied to determine whether using parametric test (student’s *t*-test) or non-parametric test (Mann–Whitney U test) to compare FGF9 expression changes between different groups. The differences between cases and controls for FGF9 mRNA/protein expressions were analyzed by the Mann-Whitney U test (Kolmogorov–Smirnov test, *p* < 0.05), whereas the difference between drug-free SCZ patients at baseline and after 8-week antipsychotics treatment for FGF9 protein levels was analyzed by the student’s *t*-test (Kolmogorov–Smirnov test, *p* > 0.05). To analyze the relationship between age, disease severity and FGF9, we used the Kendell test and Spearman test. The receiver operating characteristic (ROC) curve method was used to assess whether serum FGF9 levels could be served as a biomarker for the diagnosis and/or drug treatment response in SCZ, the accuracy of the ROC test was calculated by area under the curve (AUC). *P* < 0.05 was considered statistically significant in this study.

## Results

In this study, 57 first-episode, drug-free SCZ patients, 73 chronically medicated SCZ patients, and 111 healthy volunteers were recruited. The demographic information and clinical characteristics of all volunteers are shown in Table 1.

**TABLE 1:** Demographic and clinical characteristics of the study subjects.

	FEDF patients (*n* = 57)	ST patient (*n* = 19)	CT patients (*n* = 73)	All SCZ patients (*n* = 130)	HC (*n* = 111)
Age	29.02 ± 6.911	26.79 ± 5.950	30.14 ± 7.580	29.65 ± 7.29	28.68 ± 4.595
Gender(male/female)	25/32	9/10	36/37	62/68	60/51
Antipsychotics	–	Olanzapine/Risperidone/Palmer risperidone	Olanzapine/Risperidone/Palmer risperidone/Sodium valproate	Olanzapine/Risperidone/Palmer risperidone/Sodium valproate	–
Medicated duration	–	8 Weeks	>6 Months	–	–
FGF9 (pg/ml)	98.11 ± 56.56	163.6 ± 56.08	387.7 ± 348.6	260.7 ± 300.1	316.3 ± 132.4
PANSS total score	85.32 ± 19.70	43.21 ± 6.655	82.80 ± 21.18	83.91 ± 20.51	–
PANSS positive score	20.18 ± 6.695	9.579 ± 2.009	26.11 ± 7.417	23.51 ± 7.675	–
PANSS negative score	32.21 ± 15.57	21.32 ± 5.100	18.63 ± 6.563	24.58 ± 13.23	–

*FEDF, first-episode, drug -free; ST, short term treated; CT, chronically treated; HC, healthy control. Data were presented as mean ± SD.*

### Serum Fibroblast Growth Factor 9 Concentrations in Schizophrenia Patients

**FIGURE 1:**
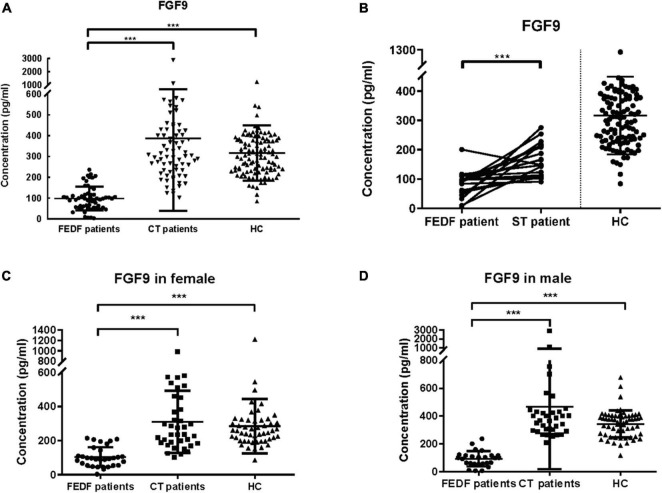
**(A)** Serum FGF9 levels were reduced in 57 first-episode, drug-free SCZ patients (Mann–Whitney *U* = 169, *P* < 0.001), but not 73 chronically treated SCZ patients (Mann–Whitney *U* = 3637, *P* > 0.05) when compared with 111 HC subjects. **(B)** Serum FGF9 levels in 19 first-episode, drug-free SCZ patients at baseline and at 8-week follow up (*t* = 4.362, *P* < 0.001). **(C)** FGF9 levels were reduced in the first-episode, drug-free SCZ female patients (Mann–Whitney *U* = 84, *P* < 0.001), but not chronically treated female SCZ patients when compared with female controls (Mann–Whitney *U* = 941, *P* > 0.05). **(D)** FGF9 levels were reduced in the first-episode, drug-free SCZ male patients (Mann–Whitney *U* = 16, *P* < 0.001), but not chronically treated male SCZ patients (Mann–Whitney *U* = 880, *P* > 0.05) when compared with male controls. FEDF, first-episode, drug-free; CT, chronically treated (long term treated); ST, short term treated (8-week); SCZ, schizophrenia; HC, healthy control. ***p < 0.001. For mean ± SD, HC: 316.3 ± 132.4; FEDF patients: 98.11 ± 56.56; CT patients: 387.7 ± 348.6; ST patients: 163.6 ± 56.08.

The results demonstrated that serum FGF9 levels were significantly reduced in 57 first-episode, drug-free SCZ patients as compared to 111 HC subjects (Figure 1A, Mann–Whitney *U* = 169, *P* < 0.001), whereas no significant difference was found between 73 chronically medicated patients and 111 controls for FGF9 levels (Figure 1A, Mann–Whitney *U* = 3637, *P* > 0.05). Furthermore, serum FGF9 levels were significantly increased in 73 chronically medicated SCZ patients relative to 57 first-episode, drug-free SCZ patients (Figure 1A, Mann–Whitney *U* = 134, *P* < 0.001). Of these SCZ patients, 19 patients from the group of drug-free SCZ patients had two evaluations: baseline and after 8-week antipsychotics treatments. The PANSS total score, PANSS positive score, and PANSS negative score the 19 patients were assessed, and the results indicated the drug treatments were effective (Table 1). The ELISA assay suggested that the serum FGF9 levels were also significantly increased in these patients after 8-week antipsychotics treatments (Figure 1B, *t* = 4.362, *P* < 0.001), suggesting that the antipsychotics up-regulated the serum FGF9 levels in the SCZ patients. In addition, we did not find gender significantly affect the dysregulation of serum FGF9 levels in the SCZ patients (Figures 1C,D).

### Blood Fibroblast Growth Factor 9 mRNA Expression in Schizophrenia Patients

**FIGURE 2:**
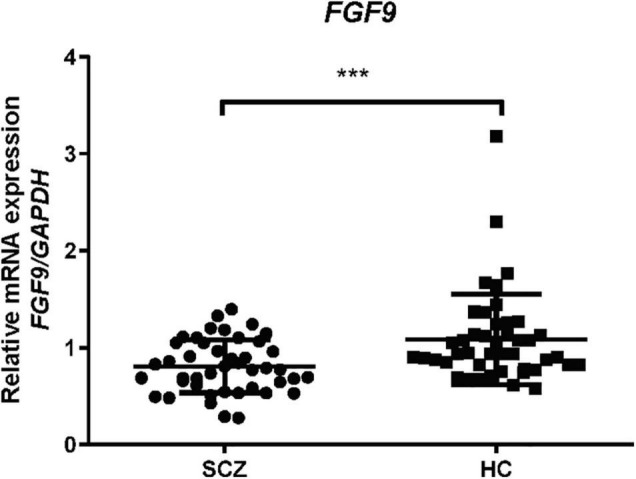
Blood *FGF9* mRNA levels were reduced in 44 drug-free SCZ patients when compared with 44 HC subjects (Mann-Whitney *U* = 578, *P* = 0.001). HC, healthy control; SCZ, schizophrenia. ***p < 0.001. For mean ± SD, HC: 1.08 ± 0.47, SCZ patients: 0.81 ± 0.28.

Next, we examined blood *FGF9* mRNA levels in the patients with SCZ, and the result from qRT-PCR showed that *FGF9* mRNA levels were significantly reduced in 44 first-episode, drug-free SCZ patients as compared with 44 controls (Figure 2, Mann–Whitney *U* = 578, *P* = 0.001).

### Relationship Between Age, Disease Severity, and Fibroblast Growth Factor 9

**FIGURE 3:**
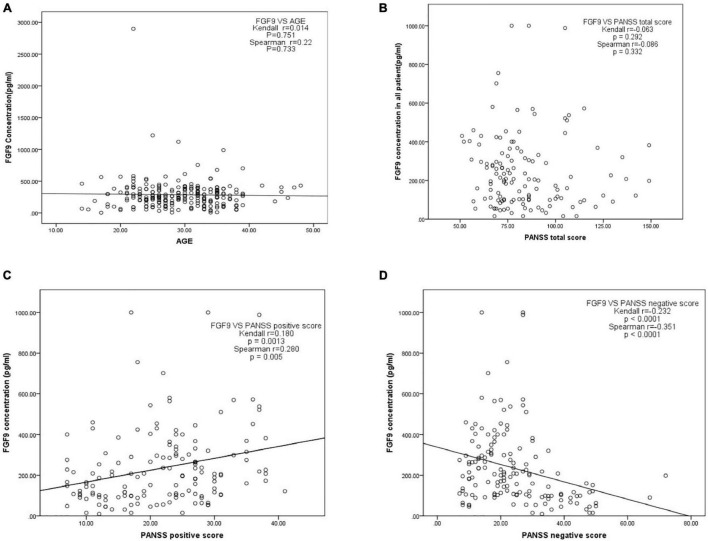
**(A)** Correlation between serum FGF9 concentration and age. **(B)** Correlation between serum FGF9 concentration and PANSS total score. **(C)** Correlation between serum FGF9 concentration and PANSS positive score. **(D)** Correlation between serum FGF9 concentration and PANSS negative score. *N* = 130.

We then investigated whether serum FGF9 levels were correlated with age and disease severity, which analyzed 130 SCZ patients. The results showed age (Figure 3A) and PANSS total score (Figure 3B) did not significantly associated with FGF9. However, there was a significantly positive correlation between serum FGF9 level and PANSS positive score in the patients (Figure 3C, Kendall *r* = 0.18, *p* = 0.0013; Spearman *r* = 0.28, *p* = 0.005). In addition, a highly negative correlation between serum FGF9 level and PANSS negative score in the patients was found (Figure 3D, Kendall *r* = −0.232, *p* < 0.001; Spearman *r* = −0.351, *p* < 0.001).

### Serum Fibroblast Growth Factor 9 as a Biomarker for Schizophrenia

**FIGURE 4:**
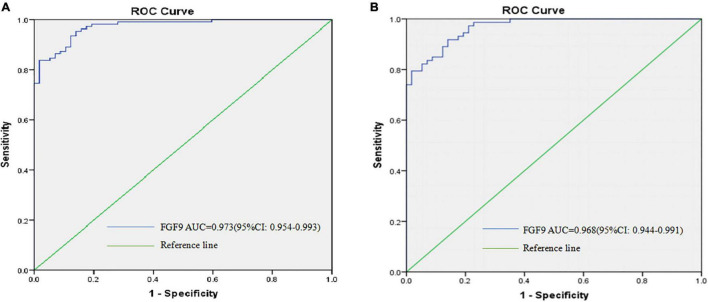
ROC curves were utilized to evaluate the accuracy of serum FGF9 concentrations to diagnose 57 first-episode, drug-free SCZ patients **(A)**, and to discriminate between 57 first-episode, drug-free SCZ patients and 73 chronically medicated SCZ patients **(B)**.

Given that the serum FGF9 levels were highly down-regulated in the patients with SCZ when compared with healthy individuals, we therefore tested whether serum FGF9 has the potential be a biomarker for SCZ. On the basis of ROC curve, the optimal cutoff value of serum FGF9 level to diagnose 57 first-episode, drug-free SCZ patients from 111 healthy individuals was projected to be 166.4 pg/ml, which yielded a sensitivity of 0.955 and specificity of 0.86 (Figure 4A and Supplementary Table 1), and the area under curve (AUC) was 0.973 (95% CI, 0.954–0.993). In addition, analyses from ROC suggested that peripheral blood FGF9 had good performance to discriminate between 57 drug-free SCZ patients and 73 chronically medicated patients, the optimal cutoff value for FGF concentration was projected to be 165.035 pg/ml with a sensitivity of 0.86 and specificity of 0.919 (Figure 4B and Supplementary Table 1), and the AUC was 0.968 (95% CI, 0.944, 0.991).

## Discussion

In this case-control study, we have included 130 patients with SCZ and 111 healthy volunteers, and demonstrated that serum FGF9 levels were highly down-regulated in the first-episode, drug-free SCZ patients. In contrast, the serum FGF9 levels in the long-term medicated SCZ patients were not significantly different when compared with healthy controls. We also performed a longitudinal study with 19 SCZ patients, and the data suggested that short-term antipsychotics treatment may increase FGF9 levels in the blood of the patients. Further analysis indicated that blood *FGF9* mRNA levels in the first-onset SCZ patients were also down-regulated. Additionally, our study indicated that gender did not significantly affect the differences of FGF9 levels between SCZ patients and controls, whereas disease severity is a confounding factor for FGF9 levels in SCZ patients. Furthermore, data from this study suggested declined PANSS positive score in SCZ patients with 8-week antipsychotics treatment, but not chronically medicated patients. It is well known that antipsychotics mainly target positive symptoms of SCZ patients, and therefore it is reasonable that PANSS positive score declined after 8-week antipsychotics treatment in the patients. However, the chronically medicated patients that we recruited were inpatients, implying that they were partially or non-response to antipsychotics for positive symptoms, and therefore it is not surprising that these patients still had high PANSS positive score. Taken together, our data provided strong clinical evidence that the onset of SCZ was accompanied by reduced blood FGF9 levels, offers a novel insight into a potential molecular pathway that confer vulnerability to the development of SCZ.

Although the data from our study revealed that the onset of SCZ was accompanied by reduced blood FGF9 levels, whether insufficiency of this growth factor in SCZ contribute to the onset of the disease is unclear. Here we hypothesize that the lack of FGF9 in SCZ contributes to the disease onset, and this is supported by the increasing evidence showing the crucial roles of FGF system in neurodevelopment and mood disorders (15). In fact, the physiological and pathological roles of FGF2 in the nervous system have been extensively studied (15). More recent *in vitro* and *in vivo* studies suggested that FGF9 was involved in the neurodevelopment and mood disorder. Results from Seuntjens et al. showed that the fate of uncommitted precursors and generation of appropriate numbers of different neurons and glia in the neocortex were regulated by neuron-to-progenitor feedback signaling, and the mechanism involved the regulation of the expression of FGF9 in the post-mitotic neurons (22). In addition, Falcone et al. indicated that FGF9 may promote astrogenesis, since they showed that the *Emx2* homeobox gene-dependent repression of FGF9 in cortico-cerebral stem cells caused the shrinkage of the astrogenic pool (23). Beside the potential role of FGF9 in neurodevelopment, FGF9 has been proposed as a novel modulator for mood disorder. This was supported by studies showing that FGF9 expression was significantly up-regulated in frontal cortex (19) and locus coeruleus (24) of patients with major depression. Consistently, animals subjected to chronic social defeat stress showed significant increase of hippocampal FGF9 expression, and administration of FGF9 increased anxiety- and depression-like behaviors in animals, whereas knocking down FGF9 expression in the dentate gyrus of the hippocampus ameliorated anxiety-like behavior (18). These results indicated that FGF9 was a modulator of negative affect for affective disorder, FGF9 thus has provided a novel therapeutic target for treatment of depression. More recently, Garrett et al. generated FGF9^*Y*162C^ mutant mice to analyze the role of FGF9 in brain functions, they discovered that the mutant mice had heightened acoustic startle reactivity and impaired social discrimination (20), therefore evidence from *in vivo* animal study suggested lacking of FGF9 activity may contribute to the etiology of SCZ. More interestingly, our results showed that antipsychotics increased serum FGF9 levels in SCZ patients. Although the molecular mechanism underlying this regulation has not been addressed in the study, one possible explanation is the involvement of brain-derived neurotrophic factor. This speculation is due to the findings that antipsychotics significantly increased brain-derived neurotrophic factor levels in SCZ patients (11), and brain-derived neurotrophic factor has been reported increasing growth factor expression *in vitro* (25). Nevertheless, future studies are necessary to explore the exact roles of FGF9 in the onset and development of SCZ.

Biomarker has been defined as “a characteristic that is objectively measured and evaluated as an indicator of normal biological processes, pathogenic processes, or pharmacological response” (26). Therefore, great efforts have been made over the last several decades to identify biomarkers for the major psychiatric diseases including SCZ (27), in hope of better understanding the etiologies of these devastating diseases, providing objective measurements for the diagnosis, prognosis and treatment response of the diseases. Although no reliable and validated biomarkers have been established for the major psychiatric diseases, potential biomarkers from blood have been extensively studied since it is easily accessible and cost-effective, and these efforts have led to some promising biomarkers for the diseases. As an example is a test called VeriPsych, which measured 51 blood analytes (small molecules and proteins), was available in 2010 from a company. This panel of biomarkers reported a specificity and sensitivity of 83% to distinguish SCZ patients from controls, and the ROC-AUC was 89% (28). However, this assay had been withdrawn from the market partly due to the specificity issue and the high cost (2500 US dollars) (27). In this study we have used a simple ELISA assay and demonstrated that blood FGF9 differentiated SCZ patients from control subjects with a sensitivity of 95.5% and specificity of 86%, and ROC-AUC of 97.3%, suggesting that FGF9 has the potential to provide excellent diagnostic accuracy for SCZ. In addition, data from our study also demonstrated that peripheral blood FGF9 had good performance to discriminate between first-onset SCZ patients and chronically medicated patients with a sensitivity of 0.86 and specificity of 0.919, suggesting FGF9 has the potential to be used as a biomarker for the treatment response of SCZ patients. However, one limitation of our study is the limited sample size of SCZ patients with 8-week antipsychotics treatment, although our analysis suggested that blood FGF9 had a reasonable performance in discriminating between first-onset SCZ patients and short-term (8-week) treated SCZ patients (data not shown). Therefore, further investigations into the peripheral blood FGF9 levels in SCZ patients are necessary to translate the potential blood biomarker into practical clinical use.

## Data Availability Statement

The raw data supporting the conclusions of this article will be made available by the authors, without undue reservation.

## Ethics Statement

The studies involving human participants were reviewed and approved by the Ethics Committee of The Third People’s Hospital of Foshan. The patients/participants provided their written informed consent to participate in this study.

## Author Contributions

X-SL and YC conceived and designed this study. X-LL, YY, and H-TW performed the experiments. X-LL, YY, YH, H-TW, and G-YC analyzed and interpreted the data. YC drafted the manuscript with critical revisions from X-LL and X-SL. All authors contributed to the article and approved the submitted version.

## Conflict of Interest

YC, YY, and YH have a patent related to this manuscript. The remaining authors declare that the research was conducted in the absence of any commercial or financial relationships that could be construed as a potential conflict of interest.

## Publisher’s Note

All claims expressed in this article are solely those of the authors and do not necessarily represent those of their affiliated organizations, or those of the publisher, the editors and the reviewers. Any product that may be evaluated in this article, or claim that may be made by its manufacturer, is not guaranteed or endorsed by the publisher.
