# Supplementary material for: Fibroblast Growth Factor 9 as a Potential Biomarker for Schizophrenia
Source: Front Psychiatry. 2022 Apr 25;13:788677. doi: 10.3389/fpsyt.2022.788677 (PMC9082542; doi:10.3389/fpsyt.2022.788677)
Supplement: Supplementary file 1 [file Table_1.pdf]

Supplementary Table 1: Data for ROC analysis

HC	FEDF patients	CT patients
172.7357	97.20992	572.3825
84.22329	92.84819	510.692
196.8754	45.49223	537.514
349.7604	53.97609	207.6042
223.6973	110.1393	451.6838
328.3029	116.5262	151.2781
258.5659	48.53808	459.7303
298.7987	143.7033	191.511
269.2946	97.74806	237.1083
215.6508	55.1645	569.7003
196.8754	6.576495	156.6425
188.8288	89.32316	170.0535
245.1549	32.43534	172.7357
199.5576	181.7693	188.8288
266.6124	10.11686	183.4644
320.2563	116.5391	298.7988
376.5824	59.89329	234.4261
280.0234	45.73182	204.922
194.1932	159.0235	218.333
304.1631	200.4896	124.4562
159.3247	88.89715	102.9986
239.7905	83.86982	199.5576
255.8837	77.59512	135.185
231.7439	63.43365	580.429
288.07	3.247428	234.4261
242.4727	208.5887	320.2563
274.659	66.97402	521.4208
261.248	151.9428	226.3795
223.6973	90.61074	274.659
172.7357	99.51825	121.774
320.4386	197.9676	296.1166
149.1413	6.787794	314.8919
415.6038	98.83731	368.5358
154.5793	99.36249	285.3878
241.5875	56.35292	280.0234
366.6617	31.57036	381.9467
543.397	62.1331	263.9302
1220.429	97.48017	263.9302
426.4798	116.5391	207.6042
497.174	108.3562	367.5641
323.1576	45.81907	289.9817
312.2816	64.85211	266.1102
194.2556	53.97609	340.7087
191.4344	121.9512	266.1102
208.3615	206.2404	286.9978
298.6396	160.0173	421.2751
287.3548	105.6372	301.9175
228.1099	102.9182	325.789
216.8251	92.04216	254.1744
236.5734	102.9182	260.1423
329.6727	102.9182	304.9014
234.4261	53.97609	307.8853
239.7905	53.97609	400.3875
245.1549	214.3974	424.259
245.1549	236.1494	755.4763
274.659	195.3644	701.7654

312.2097	105.6372	295.9496
229.0617		364.5802
263.9302		445.1466
239.7905		543.6166
218.3329		564.5042
292.9656		454.0984
337.7247		349.6605
400.3875		403.3714
373.532		400.3875
343.6926		430.2269
406.3553		331.7569
307.8853		430.2269
373.532		400.3875
388.4517		385.4678
409.3393		988.1224
400.3875		1119.517
310.8693		2897.945
382.4838		
379.4999		
292.9656		
397.4035		
325.789		
403.3714		
349.6605		
424.259		
355.6284		
388.4517		
361.5963		
334.7408		
412.3232		
373.532		
328.7729		
397.4035		
373.532		
322.8051		
612.2472		
677.8939		
415.6038		
377.5377		
116.5132		
203.5214		
391.1328		
537.959		
382.9757		
250.6794		
270.4277		
188.6132		
445.3414		
202.7192		
417.1295		
405.8448		
247.8582		
225.2887		
436.8778		
372.0997		
